# Chronic abdominal pain, appendiceal mucinous neoplasm, and concurrent intestinal endometriosis: a case report

**DOI:** 10.1186/1752-1947-6-327

**Published:** 2012-09-26

**Authors:** Takanori Kurogochi, Tetsuji Fujita, Naoko Iida, Ken Etoh, Masaichi Ogawa, Katsuhiko Yanaga

**Affiliations:** 1 Department of Surgery, The Jikei University School of Medicine, 3-25-8 Nishi-shinbashi, Minato-ku, Tokyo, Japan

**Keywords:** Chronic abdominal pain, Intestinal endometriosis, Mucinous neoplasm of the appendix

## Abstract

**Introduction:**

Although both appendiceal tumor and intestinal endometriosis have been reported as rare causes of abdominal pain, the coexistence of appendiceal mucinous neoplasm and ileal endometriosis has not previously been reported.

**Case presentation:**

A 41-year-old Japanese woman presented with a positive fecal occult blood test and a 3-year history of menstruation-related lower abdominal pain. A colonoscopy demonstrated extrinsic compression of the cecum, suggesting a mass arising from the appendix or adjacent structures. Abdominal imaging showed a 6-cm cystic mass with intraluminal thick fluids originating from the appendix. At ileocecal resection for an appendiceal tumor, a 2-cm mass in the terminal ileum was incidentally found, which was included in the surgical specimen. Microscopic examination confirmed a diagnosis of a mucinous neoplasm of the appendix with endometriosis of the terminal ileum.

**Conclusions:**

To avoid urgent surgery for subsequent serious events associated with disease progression, appendiceal tumor and intestinal endometriosis should be ruled out in patients with chronic abdominal pain.

## Introduction

Appendiceal tumor and intestinal endometriosis have been reported as rare causes of chronic abdominal pain. In a 10-year-old boy, chronic right lower abdominal pain every 3 to 6 weeks for the past year prior to presentation was attributed to a mucinous neoplasm of the appendix [1]. Surgical intervention is still the gold standard of making a tissue-based diagnosis of intestinal endometriosis, because only one third of symptomatic patients were suspected of having intestinal endometriosis preoperatively [2]. Of the symptoms related to intestinal endometriosis, chronic abdominal pain may be more common than expected. We present a case of appendiceal mucinous neoplasm with incidental intestinal endometriosis presenting with chronic abdominal pain. To the best of our knowledge, it appears that this is the first case of appendiceal mucinous neoplasm in concurrence with intestinal endometriosis.

## Case presentation

**Figure 1:**
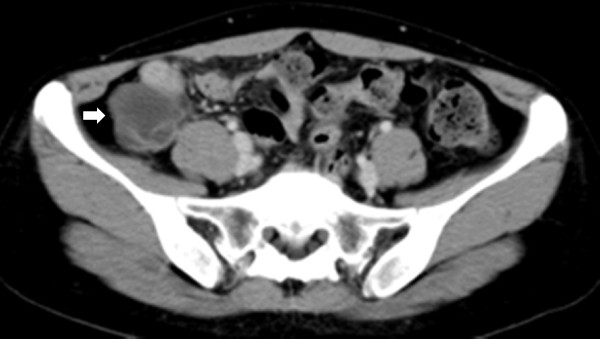
Abdominal computed tomography demonstrating a mucocele of the appendix (arrow).

**Figure 2:**
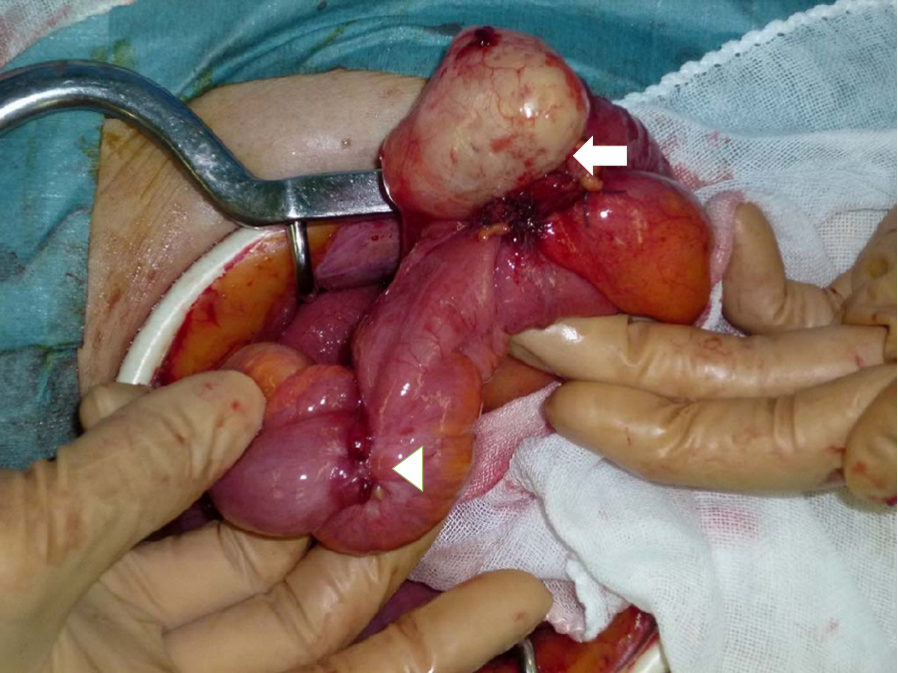
Intraoperative photograph showing a cystic tumor of the appendix (arrow) and a hard mass of the terminal ileum (arrow head).

**Figure 3:**
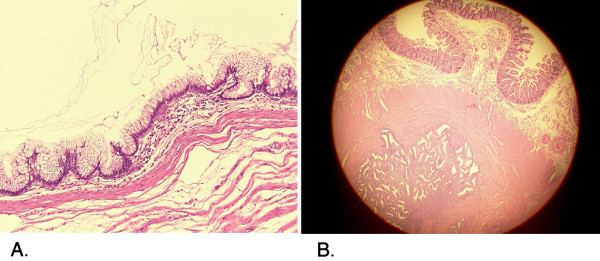
** Left figure (3A): Microscopic view of a cross-section of the appendix, whose luminal surface is circumferentially lined by low-grade dysplastic epithelium.** Right figure (3B): Microscopic view of a cross-section of the terminal ileum containing endometrial tissues in the muscularis propria layer.

A 41-year-old nulliparous Japanese woman has suffered from intermittent right lower abdominal pain for 3 years, which was sometimes related to her menstrual periods and associated with diarrhea, but settled without medical interventions. She received a colonoscopy because of a positive fecal occult blood test at a colorectal cancer screening. Colonoscopy did not detect any mucosal lesion in the colon and rectum, but demonstrated extrinsic compression of the cecum, suggesting a mass arising from the appendix or adjacent structures. Abdominal computed tomography showed a 6-cm cystic mass originating from the appendix with intraluminal thick fluids, the features of which were compatible with those of a mucinous neoplasm of the appendix (Figure 1). Magnetic resonance imaging (MRI) of the abdomen reconfirmed the findings suggestive of a mucus-producing appendiceal tumor but failed to show other intestinal lesions. All laboratory data including serum levels of carcinoembryonic antigen and carbohydrate 19–9 on admission were within normal limits. Because of the difficulty in distinguishing benign mucinous neoplasm from malignant cystadenocarcinoma based on abdominal imaging, we planned to excise the appendiceal tumor with an adequate margin and without inadvertent tumor rupture and mucous spillage during surgery. At laparotomy, a 2-cm ileal mass with serosal indentation was incidentally found at approximately 7cm from the ileocecal valve (Figure 2). There was a small myoma in the uterus, which had been identified before surgery. Otherwise, the small intestine, the mesentery, the mesenteric lymph nodes and bilateral ovaries appeared normal. Ileocecal resection including appendiceal and ileal tumors in the surgical specimen was followed by a functional end-to-end ileocecal anastomosis using linear staplers. Histopathological examination of the resected specimen revealed a mucinous neoplasm with low-grade dysplasia of the appendix (Figure 3A) and endometrioma of the terminal ileum (Figure 3B). Microscopically, intestinal endometriosis extended to the cecum and appendix but did not occlude the appendiceal lumen. The postoperative course was uneventful and she was discharged at 8 days after surgery. She was referred to a gynecologist for long-term management of endometriosis and prescribed low-dose oral contraceptive pills.

## Discussion

Approximately 25% of appendiceal mucinous neoplasms are asymptomatic and found incidentally on abdominal imaging or at surgery [3]. Rare clinical manifestations of these tumors are intestinal obstruction, intussusception, gastrointestinal bleeding and extrinsic ureteral compression. In a series of 141 patients who underwent urgent appendectomy for suspected acute appendicitis, 4 patients were found to have a mucinous neoplasm of the appendix on final pathology [4]. The classification and nomenclature of appendiceal mucinous neoplasms have not been confirmed. Classical division of these tumors into cystadenoma and cystadenocarcinoma is not necessarily suitable because Prayson and colleagues reported a series of 19 patients with pseudomyxoma peritonei, confirmed by malignant epithelium in peritoneal implants, where greater than 75% had only mucinous cystadenomas of the appendix as the primary lesion [5]. These findings were repeated in another report, in which a mucinous cystadenoma without atypical epithelium was accompanied by pseudomyxoma peritonei [6]. Recently, Pai and colleagues stratified appendiceal mucinous neoplasms into four distinct groups based on reassessment of pathologic features and long-term follow-up of 116 patients who underwent resection of appendiceal neoplasms [7]. According to their classification, the low-grade noninvasive mucinous neoplasms, all of which have been called cystadenoma, were split into three groups. Group 1 is mucinous adenoma that is confined to the appendix; Group 2 is mucinous neoplasm with low-grade dysplasia that is confined to the appendix associated with extra-appendiceal acellular mucin; and Group 3 is mucinous neoplasm with low-grade dysplasia with extra-appendiceal neoplastic epithelium. Group 4 is mucinous cystadenocarcinoma with high-grade cytology, complex architecture, or invasion. After a long-term follow-up, mucinous adenoma (Group 1) never recurred and tumors in Group 2 rarely recurred irrespective of the types of surgery, whereas in Group 3 the 5-year disease-free survival rate was 25% and in Group 4 it was 20%. Our case was classified as Group 2, low-grade mucinous neoplasm without extra-appendiceal spread. For appendiceal mucinous neoplasms, various surgical procedures such as simple appendectomy, appendectomy with partial resection of the cecum, ileocecal resection and right hemicolectomy have been performed using an either open or laparoscopic approach. The principle of surgery is complete excision of the tumor without any spillage of mucus-containing tumor cells in the peritoneal cavity. Although the size of the tumor is not likely to help in the differential diagnosis of mucinous neoplasms [7], the procedure of choice seems dependent on tumor size. We chose ileocecal resection rather than right hemicolectomy because even if the tumor is diagnosed as a cystadenocarcinoma on subsequent histopathological examination, it rarely spreads by the lymphatics [8]. Since synchronous appendiceal mucinous neoplasms and colon cancer or adenoma are not uncommon, examination of the large intestine should be done with full colonoscopy [9]. Recently, the coexistence of ileal endometriosis and carcinoid tumor of the appendix was reported [10], but a case of appendiceal mucinous neoplasm with concurrent ileal endometriosis has not been reported.

Intestinal endometriosis may be more frequent than expected, but the involvement of the terminal ileum is rare. There were 163 cases of bowel endometriosis (5.4%) among 3037 laparotomies for endometriosis, and resections of the ileum and/or cecum were performed in 11 cases, whereas a colectomy was needed in 30 cases [11]. In another report on patients with intestinal endometriosis, 72.4% had involvement of rectosigmoid, 13.5% of the rectovaginal septum, and 7% of the distal ileum [12]. The classical symptom of dysmenorrhea due to endometriosis may be absent if the bowel is exclusively involved. The superficial intestinal endometriosis may be asymptomatic, but the clinical features of a subset of intestinal endometriosis are progressive, leading to subsequent serious complications such as intestinal obstruction [13,14] and perforation [15] requiring emergency surgery.

## Conclusions

Both appendiceal tumor and intestinal endometriosis can cause chronic abdominal pain, which may be an omen of subsequent severe events associated with disease progression. Careful history taking and relevant abdominal imaging are needed when encountering a patient complaining of chronic abdominal pain.

## Consent

Written informed consent was obtained from the patient for publication of this manuscript and any accompanying images. A copy of the written consent is available for review by the Editor-in-Chief of this journal.

## Competing interests

We declare that we have no competing interests.

## Authors’ contributions

TK and TF drafted the manuscript. NI, KE and MO equally contributed to data collection and KY supervised manuscript writing. All authors read and approved the final manuscript.
